# Alcohol-abuse drug disulfiram targets pediatric glioma via MLL degradation

**DOI:** 10.1038/s41419-021-04078-9

**Published:** 2021-08-11

**Authors:** Stefanie Meier, Sandra Cantilena, Maria Victoria Niklison Chirou, John Anderson, Darren Hargrave, Paolo Salomoni, Jasper de Boer, David Michod

**Affiliations:** 1 Cancer Section, Development Biology and Cancer Programme, UCL Great Ormond Street Institute of Child Health, London, UK; 2 Centre for Therapeutic Innovation, Department of Pharmacy & Pharmacology, University of Bath, Bath, UK; 3 Nuclear Function in CNS Pathophysiology, German Center for Neurodegenerative Diseases, Bonn, Germany

**Keywords:** CNS cancer, Drug development

## Abstract

Pediatric gliomas comprise a broad range of brain tumors derived from glial cells. While high-grade gliomas are often resistant to therapy and associated with a poor outcome, children with low-grade gliomas face a better prognosis. However, the treatment of low-grade gliomas is often associated with severe long-term adverse effects. This shows that there is a strong need for improved treatment approaches. Here, we highlight the potential for repurposing disulfiram to treat pediatric gliomas. Disulfiram is a drug used to support the treatment of chronic alcoholism and was found to be effective against diverse cancer types in preclinical studies. Our results show that disulfiram efficiently kills pediatric glioma cell lines as well as patient-derived glioma stem cells. We propose a novel mechanism of action to explain disulfiram’s anti-oncogenic activities by providing evidence that disulfiram induces the degradation of the oncoprotein MLL. Our results further reveal that disulfiram treatment and MLL downregulation induce similar responses at the level of histone modifications and gene expression, further strengthening that MLL is a key target of the drug and explaining its anti-oncogenic properties.

## Introduction

Aberrant epigenetic landscapes are prevalent in cancer cells and contribute strongly to cancer development and maintenance. The idea of epigenetic therapy is to interfere with the epigenetic machinery in cancer cells to reverse aberrant patterns and to re-establish the epigenetic landscapes of healthy cells. Thereby, gene expression is normalized, and control mechanisms suppressed in malignant cells, such as cell cycle regulation or apoptosis, are restored [1]. MLL1 and MLL2 are the human homologs of Trithorax (Trx), an epigenetic regulator in Drosophila. The methylation of histone H3 lysine 4 (H3K4) residues by Trx leads to the maintenance of gene expression. The methyltransferases play a crucial role in the regulation of multiple processes during development and are implicated in the maintenance of *Hox* cluster gene expression [2]. MLL1 was found to enable cancer stem cell features and to promote cell growth and tumorigenicity in adult glioblastoma [3, 4]. MLL2 mutations were identified in 14% of Medulloblastoma patients [5]. Further, aberrant expression of multiple *Hox* cluster genes was detected in gliomas [6–8], and a *Hox* signature was associated with resistance to the chemotherapeutic agent temozolomide in pediatric glioblastoma [9].

The process of traditional drug development is not only long and costly but also inefficient. Only around 5% of cancer drugs that enter clinical trials end up being successful [10]. Since the market for drugs for rare diseases, such as pediatric cancers, is small, their development can therefore be commercially unattractive. Drug repurposing—the use of a drug for a different indication than it was originally designed and approved for—is a way to tackle the problems associated with traditional drug development [11]. The alcohol abuse drug disulfiram (Antabuse^TM^) has become of interest for drug repurposing due to its pre-clinically described anti-oncogenic properties against various human cancers [12]. Epidemiological studies revealed a trend towards reduced risk of death from cancer for patients using disulfiram as an anti-alcoholic treatment [13]. Ongoing clinical studies and literature point to the efficacy of disulfiram as a stand-alone or in combination with other drugs against metastatic liver cancer, lung cancer, prostate cancer, glioblastoma, and melanoma (http://clinicaltrials.gov).

In this report, we show that disulfiram efficiently inhibits cell proliferation of pediatric glioma cell lines and patient-derived primary cells. In addition, we propose disulfiram-induced MLL degradation as a novel mechanism of action for its cytotoxic effects in pediatric gliomas.

## Results

### MLL downregulation inhibits cell proliferation

**Fig. 1:**
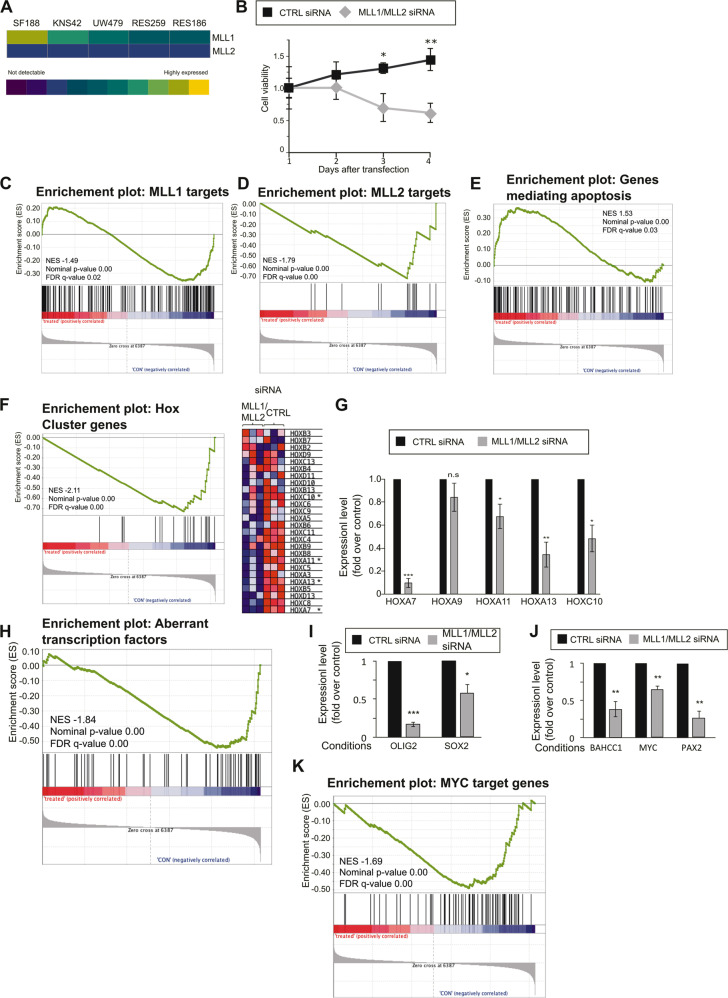
MLL downregulation inhibits cell proliferation and decreases H3K4 methylation. **A** Array data analysis derived from Bax et al. [14]. In the heat map, the relative expression levels of *MLL1* and *MLL2* are shown. **B** SF188 cells were seeded in six-well plates at a density of 0.4 × 10^6^ cells/well and transfected with 25 pmol ON-TARGETplus Control pool Non-Targeting pool (labeled C), or ON-TARGETplus SMART pool siRNA targeting *MLL1* and *MLL2* (labeled siRNA). Cell viability assays were performed on the indicated days. Data are mean ± STD; *n* = 3; ^∗^*p* < 0.05, ^∗∗^*p* < 0.01; student *t*-test. **C** GSEA showing significant negative enrichment of the WANG_MLL_TARGETS gene set after *MLL1/MLL2* downregulation in SF188 cells. The gene set comprises genes that require MLL1 for H3K4me3 and expression in mouse embryonic fibroblast cells. **D** GSEA showing significant negative enrichment of the MLL2_TARGETS gene set for *MLL1/MLL2* downregulation in SF188. The gene set comprises previously described MLL2 targets (see material and method). **E** GSEA showing significant negative enrichment of genes that mediate apoptosis by activation of caspases (MSigDB gene set: HALLMARK_APOPTOSIS) for *MLL1/MLL2* downregulation in SF188 cells. **F** GSEA showing significant negative enrichment of Hox cluster genes for *MLL1/MLL2* downregulation in SF188 cells. The gene set comprises all Hox cluster genes. The heatmap represents Hox cluster genes expression in the three independent experiments. Genes present in the core enrichment signature are highlighted in light green, genes marked with an asterisk were further analyzed by qPCR. **G**: qPCR analyses of selected HOX genes expression. Data are mean ± STD; *n* = 3; ^∗^*p* < 0.05, ^∗∗^*p* < 0.01; one-sample *t*-test. **H** GSEA showing significant negative enrichment of transcription factors with aberrant expression in glioblastoma as reported by Rheinbay et al. [20]. **I** and **J** qPCR analyses of selected transcription factors expression upon *MLL1/MLL2* downregulation. Data are mean ± STD; *n* = 3; ^∗^*p* < 0.05, ^∗∗^*p* < 0.01, ^∗∗∗^*p* < 0.001; one-sample *t*-test. **k** GSEA showing downregulation of MYC targets genes set for *MLL1/MLL2* downregulation in SF188 cells.

Gallo and colleagues [3] provided strong evidence in support of a functional MLL–HOXA10 axis in adult glioma stem-like cells. Since HOXA9 and HOXA10 have been associated with temozolomide resistance in pediatric glioma [9], we hypothesized that MLL proteins might also have a functional role in pediatric glioma. We first studied the expression levels of the two MLL proteins in five well-characterized pediatric glioma cell lines by analyzing previously published gene expression data (Supplementary Fig. 1A) [14]. Expression of *MLL2* is consistent in all five cell lines, while *MLL1* expression differs widely over the different cells lines with the highest expression being detected for the high-grade SF188 cells (Fig. 1A). We confirmed this data by western blot analysis (Supplementary Fig. 1b, MLL2 was not detectable by western blot analysis in SF188 cells with different commercially available antibodies). To explore the functional roles of MLL proteins, we downregulated *MLL1* and *MLL2* in SF188 cells using a pool of commercial siRNAs (Supplementary Fig. 1c, d). In contrast to the double downregulation, downregulation of either *MLL1* or *MLL2* alone did not lead to a significant reduction of H3K4 methylation compared with cells transfected with control siRNAs (Supplementary Fig. 1e). This indicates a functional redundancy of the two methyltransferases. We next assessed the impact of the *MLL* double downregulation on cell proliferation and detected a decrease in cell viability (Fig. 1B). Cell viability started to decline 3 days after transfection and reached a decrease of 67% compared with the control 4 days after transfection. To define the role of MLL1 and MLL2 on gene expression in pediatric glioma, we performed RNA-seq experiments. *MLL1* and *MLL2* were downregulated and RNA was extracted 72 h after transfection. As shown in Supplementary Fig. 1f, around 700 genes were downregulated upon *MLL1* and *MLL2* downregulation, while approximately 450 genes were upregulated. Gene Set Enrichment Analysis (GSEA) revealed that the *MLL* downregulation data was significantly negatively enriched for previously published target genes of MLL1 and MLL2 (Fig. 1C, D) [15–18]. Since *MLL* downregulation led to a decrease in cell viability, we next wondered if genes involved in apoptosis were modulated by *MLL* downregulation. GSEA revealed significant positive enrichment of genes mediating apoptosis showing that MLL1 and MLL2 downregulation leads to increased expression of genes involved in apoptosis (Fig. 1E). To learn more about MLL-regulated targets in SF188 cells, we next considered additional gene sets relevant to MLL. *HOX* genes are known targets of MLL proteins and are associated with resistance to temozolomide in pediatric glioblastoma [9]. GSEA revealed significant negative enrichment of the HOX gene set upon *MLL* downregulation in SF188 cells (Fig. 1F). The differential expression of five *HOX* genes was validated by qPCR (Fig. 1G). Irregular activity of transcription factors can have a major effect on gene expression and is frequently observed in cancer [19]. Rheinbay and co-workers [20] described a network of transcription factors that are aberrantly expressed in glioblastoma and promote the maintenance of cancer stem cells. We built a gene set comprising all 75 transcription factors described in this study and performed a GSEA. We observed a negative enrichment of this gene set upon *MLL1* and *MLL2* downregulation, which implies that MLL1 and/or MLL2 are involved in the positive regulation of these transcription factors, and thus might contribute to the maintenance of glioma stemness (Fig. 1H). We confirmed this observation by performing qPCR analysis on two of the transcription factors, OLIG2 and SOX2 (Fig. 1I). Both have been previously found to play important roles in glioma [21–23]. Among the genes downregulated after *MLL1* and *MLL2* downregulation in our RNA-seq data, we detected three additional transcription factors with known roles in cancer: MYC [24], PAX2 [25], and BAHCC1 [26] (data not shown). We confirmed these data by qPCR analysis (Fig. 1J) and further showed by GSEA that genes known to be regulated by MYC were negatively enriched upon *MLL1* and *MLL2* downregulation (Fig. 1K), indicating that the MYC target gene network is disrupted. Taken all together these data showed that MLL1 and MLL2 are important for glioma survival and that they regulate key pathways and expression of genes that are important for glioma biology.

### Disulfiram induces MLL degradation

**Fig. 2:**
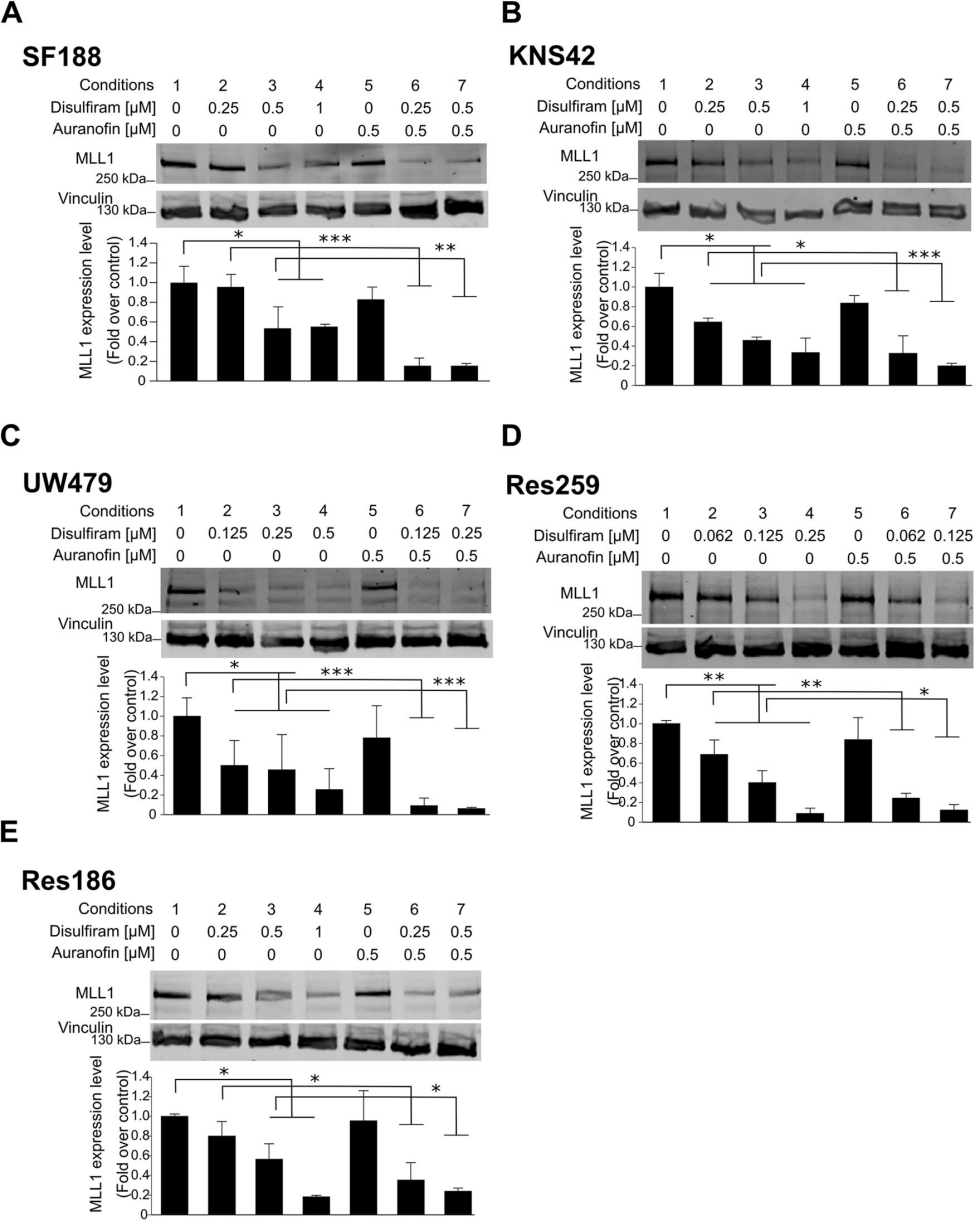
Disulfiram induces MLL degradation. **A**–**E** Top: whole-cell lysates were prepared from the indicated cell lines, cultured in the absence or presence of 0.5 mM auranofin with increasing concentrations of disulfiram for 16 h. Extracts were probed with α-MLL1 antibody and α-vinculin antibody as loading control. Bottom: the graphs represent image analysis of band intensity. Data are mean ± STD; *n* = 3; ^∗^*p* < 0.05, ^∗∗^*p* < 0.01 ^∗∗∗^*p* < 0.001; two-way analysis of variance (ANOVA) test with Bonferroni posttest.

Disulfiram has previously shown therapeutic potential for the treatment of various cancers, including glioblastoma [27–29]. When applied in combination with copper ions, disulfiram kills glioblastoma cells at physiologically achievable conditions, while the cell viability of normal cells is not affected [30]. However, the different mechanisms of action explaining the anti-oncogenic properties of disulfiram are still under investigation. We recently performed a drug screening to identify molecules that target MLL1 fusion proteins in leukemia and identified disulfiram as a candidate [31]. We showed that in addition to targeting MLL1 fusion proteins, disulfiram also induced the degradation of endogenous MLL1 in leukemia cells. To systematically study the effect of disulfiram on MLL1 in pediatric low- and high-grade glioma, we conducted a dose-response experiment on five well-characterized pediatric glioma cell lines of different grades (Supplementary Fig. 1a) [14]. Disulfiram induced a decrease in MLL1 protein levels in all tested cells, indicating that the findings from the drug screening in leukemia cells are transferable to pediatric glioma cells (Fig. 2A–E, conditions 1–4). Since disulfiram treatment did not affect *MLL1* expression (Supplementary Fig. 2A), we concluded that disulfiram modulates MLL1 at the protein level. Disulfiram contains a highly thiol-reactive functional group, which reacts with cysteine residues [32]. Both MLL1 and its paralog MLL2 contain reactive cysteines in their DNA binding CXXC domain (C is cysteine; X is any other amino acid), which is essential for the proteins’ function and association with chromatin [33]. We showed that prior to degradation, MLL1 is displaced from chromatin by disulfiram strengthening the notion that disulfiram acts on the CXXC domain of MLL (Supplementary Fig. 2b, c). We thus concluded that disulfiram was likely to induce the degradation of MLL proteins by oxidizing their CXXC domains. We next reasoned that if disulfiram-induced MLL degradation by oxidizing its CXXC domain, its action can be enhanced by inhibiting the thioredoxin reductase/thioredoxin system, an intracellular mechanism used by cells to recover from cytotoxic protein thiol oxidation [34] (see model in Supplementary Fig. 2d). Auranofin, a thioredoxin reductase inhibitor, has been already proposed to enhance disulfiram’s cytotoxic effect in ovarian cancer cells [35]. We showed that auranofin increased disulfiram-induced MLL1 degradation in pediatric glioma cells when used at a concentration of 0.5 μM (Fig. 2A–E, conditions 6 and 7), while auranofin alone had no effect on MLL1 protein levels (Fig. 2A–E, condition 5).

### Disulfiram and auranofin synergistically inhibit cell proliferation of pediatric glioma cell lines

**Fig. 3:**
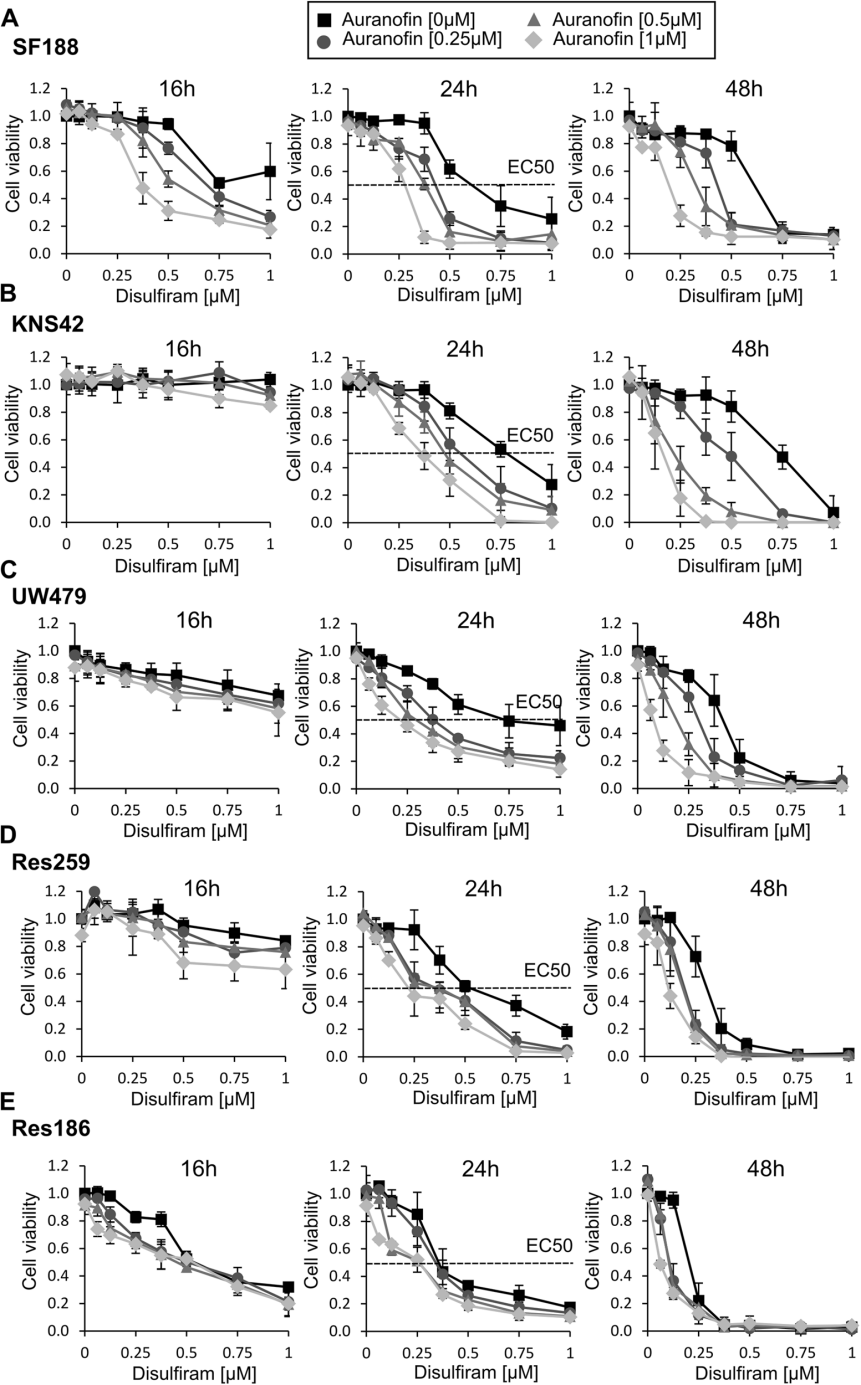
Disulfiram and auranofin synergistically inhibit cell proliferation. **A**–**E** Cells were plated in 96-wells plates. The following day cells were treated with the indicated concentrations of disulfiram in the absence or presence of increasing doses of auranofin. At the indicated time cell viability assays were performed. Data are mean ± STD; *n* = 3.

To systematically study the effect of disulfiram treatment on pediatric low- and high-grade glioma, we conducted time-course and dose-response experiments on the five pediatric glioma cell lines. We monitored the cell viability of treated cells over 48 h and observed that all pediatric glioma cells were killed within that time frame at disulfiram concentrations lower than 1 μM (Fig. 3A–E). Interestingly, we noticed that the half-maximal effective concentrations (EC50s) correlated with the cell lines’ grades (Fig. 3A–E). Low-grade glioma cell lines exhibited a lower EC50 and thus higher sensitivity to disulfiram treatment than high-grade cell lines (EC50 of 0.32–0.5 μM for Res259 and Res186, compared with EC50 of 0.62–0.75 μM for SF188, KNS42, and UW479). The same dose-response experiment was performed in combination with three increasing doses of auranofin. As shown in Fig. 3A–E, auranofin itself did not affect cell viability at the concentrations used measured by luminescent cell viability assay, however, it sensitized the cell lines to disulfiram treatment. The combination study proved that the two drugs work synergistically in pediatric glioma (Supplementary Fig. 3a–e). We next showed that disulfiram-induced cell death at 0.5 μM and that auranofin itself did not induce cell death but potentiated disulfiram-induced cell death (Supplementary Fig. 3f–j). We further confirmed that disulfiram killed SF188 cells by caspase-3 activation (Supplementary Fig. 3k).

### Disulfiram induces histone modifications

**Fig. 4:**
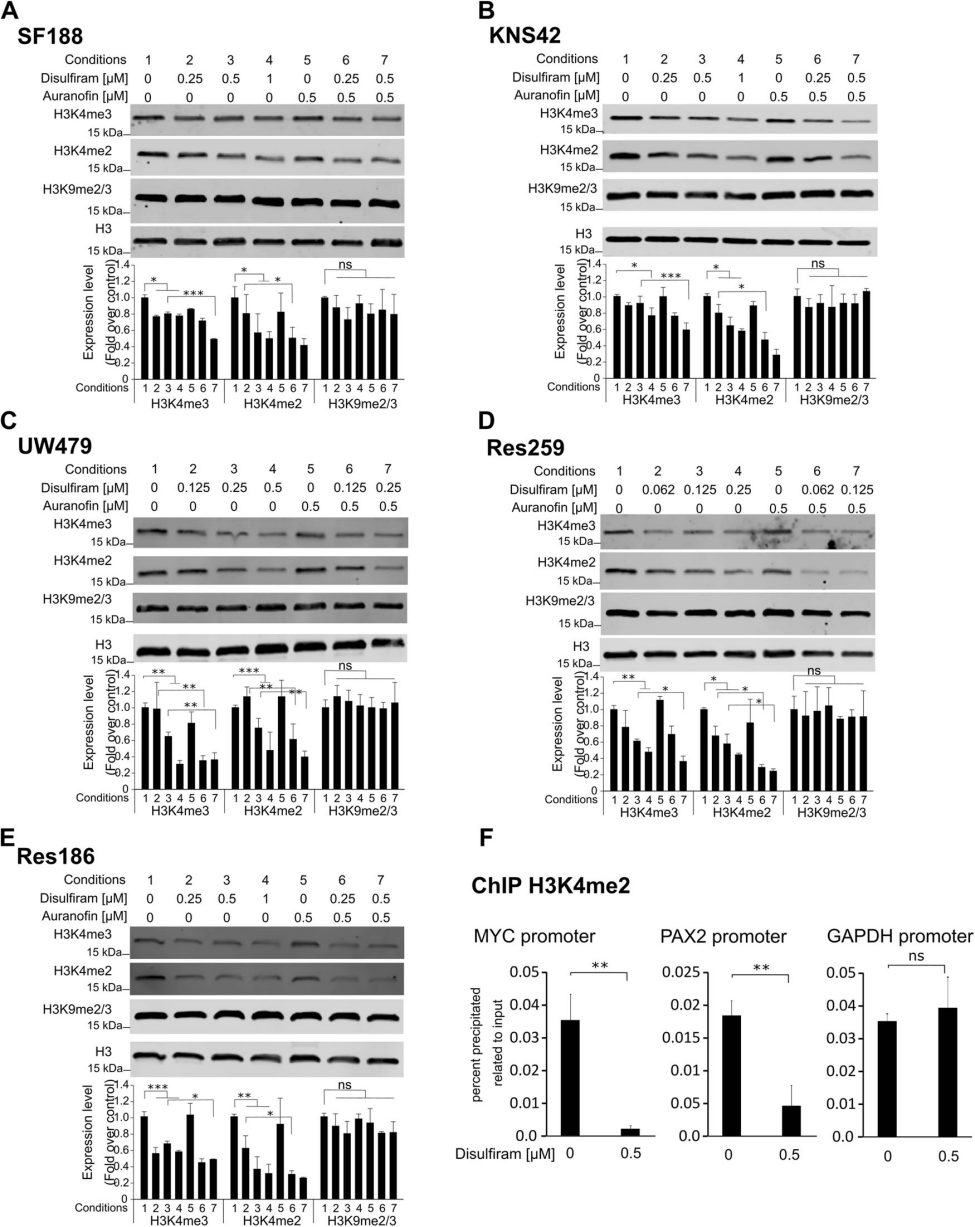
Disulfiram induces decrease of H3K4 methylation. **A**–**E** Top: whole-cell lysates were prepared from the indicated cell lines, cultured in the absence or presence of 0.5 mM auranofin with increasing concentrations of disulfiram for 16 h. Extracts were probed with α-H3K4me2, α-H3K4me3, α-H3K9me2/3 antibodies, and α-H3 antibody as loading control. Bottom: the graphs represent image analysis of band intensity. Data are mean ± STD; *n* = 3; ^∗^*p* < 0.05, ^∗∗^*p* < 0.01 ^∗∗∗^*p* < 0.001; two-way analysis of variance (ANOVA) test with Bonferroni posttest. **F** Chromatin immunoprecipitation analysis of H3K4me2 enrichment at promoter regions of MYC and PAX2. ChIP was performed using chromatin from SF188 left untreated or treated for 16 h with 0.5 µM disulfiram. Data are mean ± STD; *n* = 3; ^∗∗^*p* < 0.01, student *t*-test.

The histone methyltransferases MLL1 and MLL2 are responsible for the addition of methyl groups to the histone H3 tail amino acid lysine 4 (H3K4) [36]. H3K4 can be mono-, di-, or trimethylated, with both di- and trimethylation being associated with promoter regions of active transcription [37]. To assess whether the treatment with disulfiram alone or in combination with auranofin affects the methyltransferase activity, we studied the level of H3K4 methylation in treated cells. As shown in Fig. 4A–E conditions 1–4, disulfiram efficiently decreased the level of H3K4 dimethylation (H3K4me2) in all cell lines, as well as the level of H3K4 trimethylation (H3K4me3), although to a lesser extent. When disulfiram was used in combination with auranofin, the decrease in both di- and trimethylation was even greater (Fig. 4A–E, compare conditions 2 with 6 and conditions 3 with 7), while auranofin alone did not affect the methylation status of H3K4 (Fig. 4A–E, condition 5). None of the treatments affected the level of H3K9 methylation (Fig. 4A–E), indicating that disulfiram specifically interfered with the histone modifications associated with MLL1 and MLL2 activity. We next assessed the level of H3K4 dimethylation upon disulfiram treatment at the promoter region of MYC and PAX2, two genes affected by MLL downregulation (Fig. 1J). We showed that disulfiram-induced a marked decrease of H3K4me2 at both promoter regions of *MYC* and *PAX2* while not affecting the promoter region of GAPDH (Fig. 4F). Thus, indicating that disulfiram-induced a decrease of H3K4 methylation at distinct regions rather than affecting the overall level of H3K4me2.

### Disulfiram-induced MLL degradation inhibits proliferation of high-grade glioma patient-derived primary cells

**Fig. 5:**
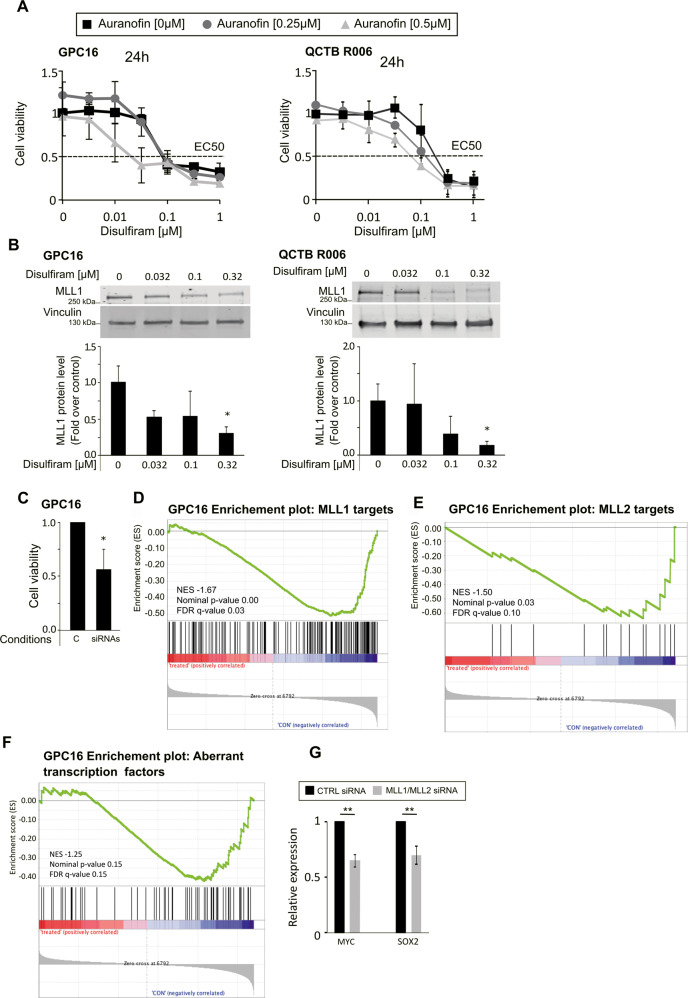
Disulfiram inhibits cell proliferation and induces MLL degradation in patient-derived primary high-grade glioma cells. **A** Cells were plated in 96-wells plates. The following day cells were treated with the indicated concentrations of disulfiram in the absence or presence of increasing doses of auranofin. At the indicated time cell viability assays were performed. Data are mean ± STD; *n* = 3. **B** Top: whole-cell lysates were prepared from the indicated cells, cultured in the absence or presence of increasing concentrations of disulfiram for 16 h. Extracts were probed with α-MLL1 antibody and α-vinculin antibody as loading control. Bottom: the graphs represent image analysis of band intensity. Data are mean ± STD; *n* = 3; ^∗^*p* < 0.05; two-way analysis of variance (ANOVA) test with Bonferroni posttest. **C** GPC16 cells were seeded in six-well plates at a density of 0.4 × 10^6^ cells/well and transfected with 25 pmol ON-TARGETplus Control pool Non-Targeting pool (labeled C), or ON-TARGETplus SMART pool siRNA targeting *MLL1* and *MLL2* (labeled siRNA). Cell viability assays were performed 3 days after transfection. Data are mean ± STD; *n* = 3; ^∗^*p* < 0.05; one-sample *t*-test. **D**, **E**, and **F** same experiment as Fig. 1 but with GPC16 cells. **G** qPCR analyses of selected transcription factors expression upon *MLL1/MLL2* downregulation in GPC16 cells. Data are mean ± STD; *n* = 3; ^∗∗^*p* < 0.01; one-sample *t*-test.

To confirm our findings in a cell culture model that closely represents the human in vivo patient condition, we used two primary pediatric patient-derived high-grade glioma stem cells, GPC16 and QCTB R006. First, we conducted a disulfiram dose-response experiment in the absence or presence of increasing doses of auranofin, and we monitored the viability of treated cells over 24 h. We observed that both primary cells were killed within that time frame at disulfiram concentrations lower than 0.1 μM for GPC16 and 0.5 μM for QCTB R006 (Fig. 5A). In comparison with the high-grade glioma cell lines SF188 and KNS42, the primary cells exhibited lower EC50s (SF188 EC50: 0.62 μM, KNS42 EC50: 0.75 μM (Fig. 2A–E), GPC16 EC50: 0.1 μM, QCTB R006 EC50: 0.3 μM (Fig. 5A)) and thus higher sensitivity to disulfiram. However, the synergistic effect of the disulfiram/auranofin combination treatment on primary cells was not as strong as on high-grade glioma cell lines (synergy scores of 8.4 and 15.1 for GPC16 and QCTB R006 respectively (Supplementary Fig. 4a), compared with 24.5 and 19.5 for SF188 and KNN42 respectively (Supplementary Fig. 3a, b)). The two primary cells also showed a decrease in MLL1 protein levels upon treatment with disulfiram (Fig. 5B). We next tested if downregulation of MLL proteins had a similar effect on cell proliferation as observed for the SF188 cell line. The same siRNA pools were used for the downregulation of *MLL1* and *MLL2* in the primary cells as previously used in SF188 cells. We achieved a significant downregulation of *MLL1* and *MLL2* gene expression in GPC16 cells (Supplementary Fig. 4b) that is comparable to what we achieved in SF188 cells (Supplementary Fig 1d). However, the downregulation was not successful in QCTB R006 cells (data not showed), probably due to low transfection efficiency of these cells with the current protocol. We next assessed the effect of *MLL1* and *MLL2* downregulation on cell proliferation in GPC16. As shown in Fig. 5C, *MLL1* and *MLL2* downregulation decreased the viability of GPC16 cells within 3 days by 50%. We performed RNA-seq experiments to define the role of MLL1 and MLL2 on gene expression in GPC16 cells and noticed several similarities between the effect of the *MLL* downregulation in GPC16 and in SF188. As in SF188, it also led to the downregulation of previously published target genes of MLL1 and MLL2 (Fig. 5D, E), as well as genes that are part of the network of transcription factors that are aberrantly expressed in glioblastoma in GPC16 cells (Fig. 5F). We further showed that *SOX2* and *MYC* were also negatively regulated upon *MLL1* and *MLL2* downregulation, although the downregulations were less marked than in SF188 cells (Fig. 5G). In addition, we also noticed some differences compared with the SF188 cells. *HOX* genes and *PAX2* were not expressed at levels detectable by RNA-seq or qPCR analysis in GPC16 cells (data not shown), indicating that the expression of certain development-related transcription factors may vary between cell lines and primary cells, or generally between glioma cells. Finally, we did not observe an effect of *MLL* downregulation on the expression of *OLIG2* and *BAHCC1* (Fig. 5G, H).

Altogether, these results indicate that primary patient-derived cells are more sensitive to disulfiram compared with glioma cell lines. The findings further suggest that disulfiram-induced MLL protein degradation contributes to the inhibition of cell proliferation of primary patient-derived cells. Finally, these data showed that MLL1 and MLL2 generally regulate similar pathways and gene expression in SF188 and GPC16 cells, although there are some differences.

### Disulfiram induces transcriptional changes that mimic *MLL* downregulation

**Fig. 6:**
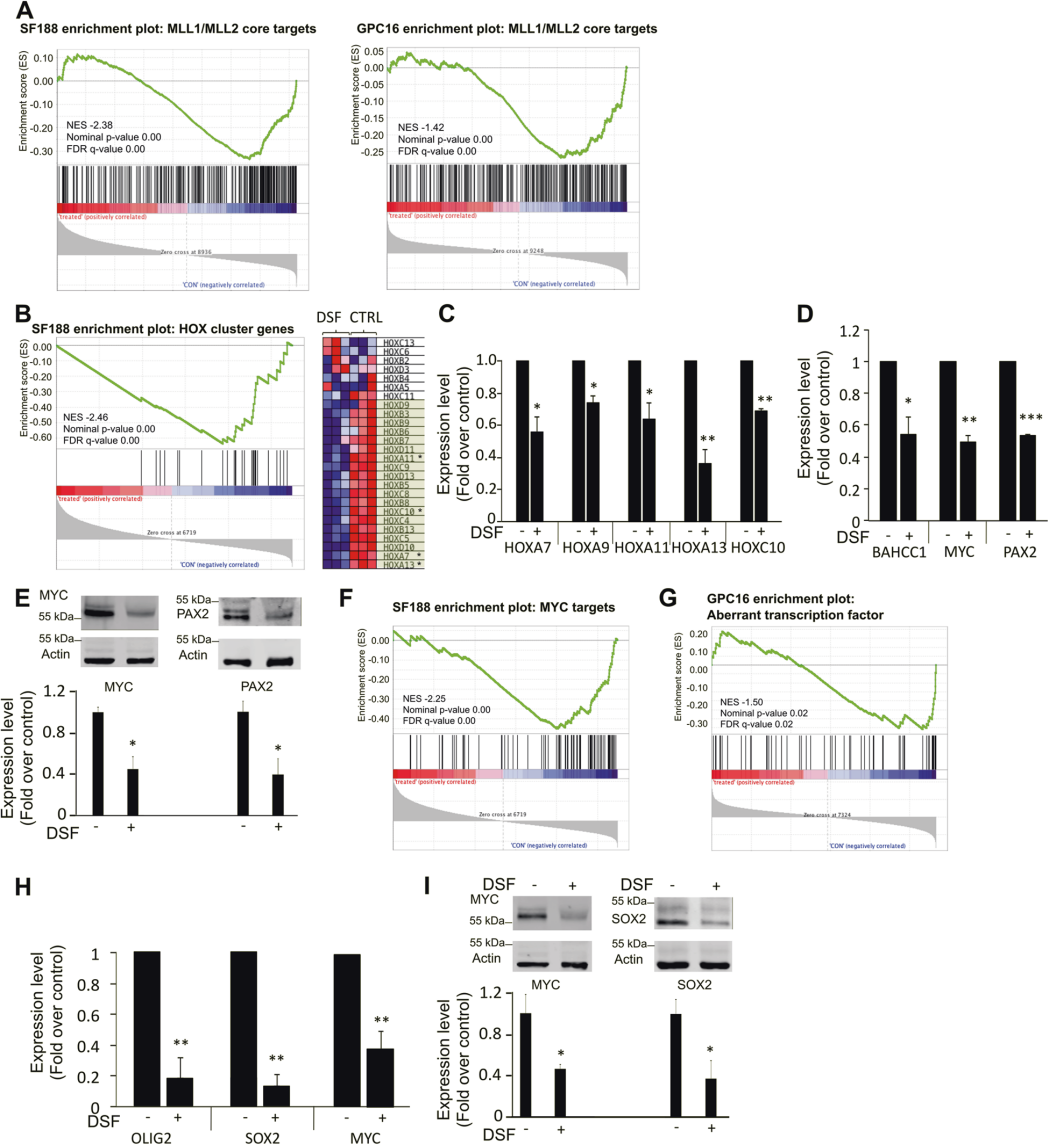
Changes in SF188 and GPC16 gene expression following disulfiram treatment. **A** and **B** GSEA showing downregulation of *MLL1/MLL2* core genes set (genes downregulated upon *MLL1/MLL2* downregulation) in disulfiram-treated SF188 and GPC16 cells. **C** GSEA showing downregulation of HOX cluster genes set (gene set including all 39 human Hox cluster genes) in disulfiram-treated SF188 cells. The heatmap is part of the GSEA. It represents Hox cluster genes expression in disulfiram-treated (DSF) and untreated cells (CTRL) with three replicates each. Genes present in the core enrichment signature are highlighted in green, genes marked with an asterisk were further analyzed. **C** qPCR analyses of HOX genes expression in disulfiram-treated SF188 cells. Data are mean ± STD; *n* = 3; ^∗^*p* < 0.05, ^∗∗^*p* < 0.01; one-sample *t*-test. **D** qPCR analyses of transcription factors expression upon disulfiram treatment. Data are mean ± STD; *n* = 3; ^∗^*p* < 0.05, ^∗∗^*p* < 0.01, ^∗∗∗^*p* < 0.001; one-sample *t*-test. **E** Western blot analysis of MYC and PAX2 expression level upon disulfiram treatment. Data are mean ± STD; *n* = 3; ^∗^*p* < 0.05, *t*-test. **F** GSEA showing downregulation of MYC targets genes set (genes upregulated upon MYC expression) in disulfiram-treated SF188 cells (MSigDB gene set: SCHUHMACHER_MYC_TARGETS_UP). **G** GSEA showing downregulation of aberrant transcription factor set (75 transcription factors with aberrant expression in glioblastoma as reported by Rheinbay et al.) in disulfiram-treated GPC16 cells. **H** qPCR analyses of *OLIG2*, *SOX2*, and *MYC* expression in disulfiram-treated GPC16 cells. Data are mean ± STD; *n* = 3; ^∗∗^*p* < 0.01; one-sample *t*-test. **I** Western blot analysis of SOX2 and MYC expression level upon disulfiram treatment. Data are mean ± STD; *n* = 3; ^∗^*p* < 0.05, *t*-test.

Our previous experiments showed that disulfiram treatment leads to *MLL* degradation and a decrease in H3K4 methylation. To assess whether disulfiram treatment also affects the expression of genes epigenetically regulated by MLL proteins, we first used RNA-seq to identify genes differentially expressed upon the downregulation of *MLL1* and *MLL2* with siRNA. Genes identified as significantly downregulated under this condition in both the cell line SF188 and the glioma stem cells GPC16 were used to build a gene set called MLL1_MLL2_core (Supplementary Table 1). Since MLL1 and MLL2 are primarily known to be involved in the positive regulation of gene expression, we focused on genes that showed a decrease in expression upon MLL downregulation. RNA-seq of SF188 and GPC16 cells treated with disulfiram, as well as untreated cells showed that the drug treatment led to differential expression of around 8000 genes (Supplementary Fig. 5a). GSEA revealed significant negative enrichment for the MLL1_MLL2 core genes set in disulfiram-treated SF188 and GPC16 cells, demonstrating that genes regulated by MLL1 and MLL2 are affected by disulfiram treatment in both glioma cell line and primary cells (Fig. 6A). This indicates that the decrease in MLL1 and MLL2 protein levels upon disulfiram treatment, and thus the reduced methyltransferase activity, leads to the downregulation of genes normally positively regulated by the two methyltransferases. To learn more about the effect of disulfiram on MLL-regulated targets, we next considered additional gene sets relevant to MLL. GSEA revealed significant negative enrichment of Hox cluster gene set upon disulfiram treatment in SF188 cells, showing that the treatment leads to the downregulation of Hox cluster genes (Fig. 6B). Their differential expression upon disulfiram treatment was validated by qPCR (Fig. 6C). While *HOXA9* expression was not detectable by RNA-seq, it was detectable in disulfiram-treated and untreated cells using qPCR (Fig. 6C). These findings show that disulfiram treatment affects the expression of additional genes regulated by MLL1 and MLL2, and that disulfiram can be used to disrupt the expression of *HOX* genes, which are frequently associated with tumorigenesis in high-grade glioma [3]. Among the genes downregulated upon *MLL1* and *MLL2* downregulation, we detected *BAHCC1 MYC* and *PAX2* (Fig. 1J). Using qPCR we confirmed that the three genes were also downregulated in disulfiram-treated SF188 cells (Fig. 6D). We confirmed for MYC and PAX2 that the decrease in gene expression resulted in protein downregulation (Fig. 6E). Further, GSEA revealed a significant negative enrichment of MYC target genes in treated SF188 cells (Fig. 6F), showing that disulfiram treatment not only disrupts the expression of *MYC* but also leads to the downregulation of its target genes. Similarly, we detected that disulfiram treatment affects the expression of cancer-associated transcription factors in the patient-derived GPC16 cells (Fig. 6G). We further validated this finding by analyzing the expression of some of these transcription factors by qPCR and western blot. A reduction of over 80%, 85%, and 60% was detected for *SOX2*, *OLIG2*, and *MYC* respectively at the level of gene expression (Fig. 6H), and a reduction of over 50% was detected for MYC and SOX2 at the protein level (Fig. 6I).

## Discussion

Various mechanisms have been proposed to explain the cytotoxic effect of disulfiram on cancer cells [29, 30, 38]. Disulfiram has been shown to induce proteasome inhibition that leads to the accumulation of misfolded proteins and possible toxic protein aggregates [28]. More recently, disulfiram’s tumor-suppressing effects have been attributed to its action on the protein NPL4 and the resulting accumulation of ubiquitinated proteins [13]. We proposed here, that, in addition to the aforementioned targets, disulfiram-induced MLL degradation contributes to its anti-oncogenic properties. Repurposing of disulfiram for cancer treatment has already been studied in several cancers and multiple clinical trials have been done to validate its potential use as a drug to fight cancer. MLL1 has been associated with many types of cancer [4, 39–41]. In addition, MLL1 plays a critical role in tumor growth and angiogenesis and its knockdown suppresses tumor growth in vivo. Our data indicate that disulfiram targets MLL1 [42]. It is thus of interest to further test the efficacy of disulfiram in MLL1-, and potentially MLL2-driven cancers. We showed that disulfiram kills pediatric glioma stem cells at low concentrations and cell lines at slightly higher concentrations. We found that the drug’s efficiency can be increased by the addition of auranofin and that the synergistic effect of the two drugs is more pronounced in differentiated cells compared to undifferentiated cells. This indicates that disulfiram alone efficiently kills glioma cancer stem cells and in combination with auranofin can kill more differentiated cancer cells, suggesting that disulfiram is efficient against heterogeneous lineages of cancer cells. In addition to induced MLL1 degradation, disulfiram also interacts with previously proposed targets of the drug in pediatric glioma. This shows that disulfiram’s mode of action in pediatric glioma cells is multi-modal and thus advantageous over single target molecules, which are often prone to therapy resistance.

Taken altogether, our study showed that disulfiram kills pediatric glioma cells at doses relevant to human treatment, hence adding pediatric glioma to the list of cancers that potentially can be treated with disulfiram. The current treatment of pediatric is primarily based on chemotherapeutic compounds, and often has serious side effects in children. Well-tolerated drugs such as disulfiram and auranofin are thus of particular interest for the treatment of this disease.

## Materials and methods

### Cell culture

GPC16 and QCTB ROS 006 were maintained in NeuroCult™ NS-A Basal medium (Human, STEMCELL™ technologies), supplemented with penicillin (100 units/ml), streptomycin (100 μg/ml), epidermal growth factor (murine EGF, 20 ng/ml, PEPROtech), fibroblast growth factor (human FGF-basic, 20 ng/ml, PEPROtech), platelet-derived growth factor (PDGF-AB, 20 ng/ml, PEPROtech) and NeuroCult™ NS-A Proliferation Supplement (Human, STEMCELL™ technologies). QCTB ROS 006 was obtained from A. Moore, GPC16 cells were established from a patient treated at Great Ormond Street Hospital diagnosed with high-grade glioma. All patient samples were collected under full Research Ethics Committee approval. The pediatric glioma cell lines were obtained from C. Jones and were cultured in Dulbecco’s Modified Eagle’s Medium/Nutrient Mixture F-12 (DMEM/F-12, SIGMA Life Science) supplemented with penicillin (100 units/ml), streptomycin (100 μg/ml), and 10% Fetal Bovine Serum (FBS, Gibco by Life Technologies). All cells were grown as monolayers at 37 °C in 5% CO_2_. All cells were regularly tested for mycoplasma and purity of the culture by STR profiling.

### Drug treatment

Prior to their use, disulfiram (Sigma-Aldrich) and auranofin (Tocris) were dissolved in DMSO. The dissolved drugs were added to the cells for treatment, the same quantity of DMSO (without drug) was added to the control samples. CuCl_2_ was added to all samples of experiments involving disulfiram (also to the control sample) to reach a final concentration of 1 μM.

### Cell viability assay

The CellTiter-Glo Luminescent Cell Viability Assay (Promega) was used according to the manufacturer’s protocol. Cells were lysed on an orbital shaker for 2 min. After stabilizing the luminescent signal for 10 min at room temperature, the content of the wells was transferred to a white v-bottom Greiner Bio-One 96-well. Luminescence was measured using a TECAN infinite M200PRO plate reader.

### Mitochondrial membrane potential assay

JC-10 Mitochondrial Membrane Potential Assay (Abcam, ab112134) was used according to the manufacturer’s protocol. Luminescence was measured using a TECAN infinite M200PRO plate reader.

### Caspase-3 activity assay

Caspase-3 Activity Assay kit (Abcam, ab252897) was used according to the manufacturer’s protocol. Luminescence was measured using a TECAN infinite M200PRO plate reader.

### Histone extraction for western blot analysis

Histones were extracted by adapting the histone extraction protocol for western blot from Abcam. Cells were resuspended in Triton Extraction Buffer (TEB) and lysed for 10 min at 4 °C. The lysate was centrifuged for 10 min at 6500 x *g* at 4 °C to collect the nuclei. The supernatant was discarded, and the pellet was washed in TEB and centrifuged again using the same conditions. The pellet was resuspended in 0.2 M HCl (4 × 10^7^ nuclei/ml) and the histones were extracted overnight at 4 °C. The sample was centrifuged for 10 min at 6500 x *g* at 4 °C and the supernatant was collected. 2 M NaOH was used to neutralize the supernatant. Reducing Sample Buffer was added to the extracts (1:1). The extracts were stored at −20 °C.

### Western blot

Proteins were resolved on sodium dodecyl sulphate–polyacrylamide gel electrophoresis and transferred to nitrocellulose membrane. Membranes were blocked for one hour in PBS 5% nonfat dry milk at room temperature and primary antibodies incubated O/N at 4 °C. The primary antibodies were detected by DyLight 680 or 800 conjugated secondary antibodies (LI-COR) diluted 1:5000 in PBS Tween 0.1% and 5% nonfat dry milk and subsequently visualized with the Odyssey® Infrared Imaging System (LI-COR). The antibodies specific for MLL1 (Millipore, 05-764), Vincullin (Abcam, ab129002), H3K4me2 (Abcam, ab32356), H3K4me3 (Abcam, ab8580), H3K9me2/3 (Cell Signalling, 5327), H3 (Abcam, ab10799), MYC (Abcam, ab32072), PAX2 (Abcam, ab79389) SOX2 (Abcam, ab133337) were diluted 1:1,000 in PBS Tween 0.1% and 5% nonfat dry milk O/N at 4 °C.

### RNA isolation, RT-PCR, and quantitative real-time PCR analysis

Total RNA was prepared using an RNeasy kit (Qiagen). For reverse transcription, complementary DNA (cDNA) Reverse Transcription Kit (Applied Biosystems) was used and quantitative real-time PCR was performed using Maxima SYBR Green/ROX qPCR Master mix (ThermoFisher Scientific). Relative abundance of the specific mRNAs was normalized to Beta-Actin mRNA. Primer sequences: ACTB: tccgtgtggatcggcggctcca, ctgcttgctgatccacatctg. BAHCC1: catcatcacatccgaaccag, tgaggtggatggatcatttg. HOXA7: ctggatgcggtcttcagg, ggtagcggttgaagtggaac. HOXA9: gcgccttctctgaaaacaat, cgctttttccgagtggag. HOXA11: cggcagcagaggagaaag, gtataggggcagcgctttt. HOXA13: cctctggaagtccactctgc, ggtataaggcacgcgcttc. HOXC10: aggagagggccaaagctg, agccaatttcctgtggtgtt. LDHA: ccgttacctaatgggggaaa, gcaacattcattccactcca MLL1: gtcgaccgttgccttctg, gggtgataaggaagaggtactgtg. MLL2: ggagaaccagaccattgtgc, ttctgaatgggcgagtgg. MYC: gaccagctggagatggtgac, ggtcgcagatgaaactctgg. OLIG2: tcctcaaatcgcatccaga, gaaaaaggtcatcgggctct. PAX2: cagcgtctcttccatcaacag, gtgctgggaacaatggtgt SOX2: ttgctgcctctttaagactagga, taagcctggggctcaaact.

### Chromatin Immunoprecipitation

In all, 0.5 × 10^6^ cells were crosslinked in 1% formaldehyde. Chromatin immunoprecipitation was performed using Imprint Chromatin Immunoprecipitation Kit (Sigma-Aldrich, #CHP1) per manufacturer’s instructions. Sonication was performed using Diagenode bioruptor with the following setup: 30 s on 50% power, 30 s off, 10 cycles. For quantitative ChIP, DNA amplification was performed with Maxima SYBR Green/ROX qPCR Master mix (ThermoFisher Scientific). Percent input was calculated with the formula 100 × 2^(Ctadjusted input − CtIP)^. Input DNA Ct was adjusted from 1% to 100% equivalent by subtracting 6.644 Cts (Log_2_100) from original Ctinput. Two micrograms of MLL1 (Bethyl, A700-010) or H3K4me2 (Abcam, ab32356) antibodies were used. Primers sequence are MYC promoter: actcacaggacaaggatgcg, gcgcgcctaccattttcttt. PAX2 promoter: caagtcatccatctcccggc, tcccggtgtgtgtctctcta. GAPDH promoter: caattccccatctcagtcgt, tagtagccgggccctacttt.

### MLL1 and MLL2 downregulation

Small interfering RNAs (siRNA) were purchased from Dharmacon (MLL1: ON-TARGETplus SMART pool Human MLL, MLL2: ON-TARGETplus SMART pool Human MLL4). ON-TARGETplus Control pool Non-Targeting pool was used for control. Cells were plated and transfected on the following day using OPTI-MEM (gibco by Life Technologies) and Lipofectamine RNAiMAX (Invitrogen) according to the manufacturer’s instructions. Nine microliters Lipofectamine was added to 150 μl OPTI-MEM. The appropriate amount of siRNA (30 pmol for SF188, 300 pmol for GPC16 and QCTB R006) was diluted in 150 μl OPTI-MEM. The two solutions prepared were mixed and incubated for 5 min. Two-hundred fifty microliters of this mix was added to the cells.

### RNA-seq

Library preparation, RNA sequencing, and reads alignment were conducted by the UCL genomics team. RNA integrity was confirmed using the Agilent 2200 TapeStation System (Agilent technologies). Two-hundred nanograms of total RNA were processed using the NEBNext RNA Ultra II kit with Poly A + selection (p/n E7760 & E7490) according to manufacturer’s instructions. Oligo dT beads were used to pull down polyadenylated mRNA transcripts and isolate them from total RNA. Chemical fragmentation was applied to the purified mRNA. The fragments were primed with random hexamers, and strand-specific first-strand cDNA was generated 80 using Reverse Transcriptase and Actinomycin-D. dUTP instead of dTTP was used for the synthesis of the second cDNA strand to mark it. To prevent self-ligation and adapter dimerization the cDNA was then “A-tailed” at the 3’ end. Truncated Y adaptors with a T overhang were ligated to the A-Tailed cDNA. The ligated fragments were amplified with 14 cycles of PCR. The primers used contain a 6 bp Index sequence that allows each library to be identified. Only the first-strand cDNA is amplified since the taq polymerase employed in the PCR is unable to read through uracil. This makes the library strand-specific. Qubit and Bioanalyser fragment analyses were used to calculate equimolar quantities for the libraries to be multiplexed in the same run. Sequencing was performed on the NextSeq 500 instrument (Illumina, San Diego, US) using a 43 bp paired-end run. This resulted in > 15million reads per sample. Illumina’s bcl2fastq Conversion Software v2.19 was used to demultiplex and convert the run data to fastq files. These were aligned to the human genome UCSC hg38 using RNA-STAR 2.5.2b and deduplicated using Picard Tools 1.79. FeatureCounts was used to count the reads per transcript and differential expression assessed with the BioConductor package SARTools, a DESeq2 wrapper. The annotation and sequences were obtained from Illumina iGenomes. RNA-seq data can be accessed via GEO Profiles (GSE139016).

### Gene set enrichment analysis (GSEA)

RNAseq data sets consisted of all genes with a mean count above 10 in the MLL1/MLL2 knockdown or disulfiram-treated sample and the control sample. The data sets were analyzed for the enrichment of gene sets using the Gene Set Enrichment Analysis (GSEA) software from the Broad Institute. Each gene set included between 15 and 500 genes and can be found in the Molecular Signature Database (MSigDB) or in appendix 1. The following settings were applied for the GSEA: numbers of permutations: 1000; permutation type: gene set. A gene set was considered significantly enriched if the nominal *p*-value 81 was <0.05. According to GSEA results with a false-discovery rate (FDR) below 25% can indicate a trend. Thus, results with a p-value above 0.05 and an FDR below 25% were considered as potential findings. Correlation between data sets was detectable as a positive enrichment score, while inverse correlation showed as a negative score.

### Statistical analysis

Statistical analyses were performed using graphpad Prism software.

## Supplementary information

Supplementary fig.1

Supplementary fig.2

Supplementary fig.3

Supplementary fig.4

Supplementary fig.5

Supplementary Table1

## References

[1] Yoo CB, Jones PA (2006). Epigenetic therapy of cancer: past, present and future. Nat Rev Drug Discov.

[2] Schuettengruber B, Bourbon H-M, Di Croce L, Cavalli G (2017). Genome regulation by polycomb and trithorax: 70 years and counting. Cell.

[3] Gallo M, Ho J, Coutinho FJ, Vanner R, Lee L, Head R (2013). A tumorigenic MLL-homeobox network in human glioblastoma stem cells. Cancer Res.

[4] Heddleston JM, Wu Q, Rivera M, Minhas S, Lathia JD, Sloan AE (2012). Hypoxia-induced mixed-lineage leukemia 1 regulates glioma stem cell tumorigenic potential. Cell Death Differ.

[5] Parsons DW, Li M, Zhang X, Jones S, Leary RJ, Lin JC (2011). The genetic landscape of the childhood cancer medulloblastoma. Science..

[6] Costa BM, Smith JS, Chen Y, Chen J, Phillips HS, Aldape KD (2010). Reversing HOXA9 oncogene activation by PI3K inhibition: epigenetic mechanism and prognostic significance in human glioblastoma. Cancer Res.

[7] Duan R, Han L, Wang Q, Wei J, Chen L, Zhang J (2015). HOXA13 is a potential GBM diagnostic marker and promotes glioma invasion by activating the Wnt and TGF-β pathways. Oncotarget..

[8] Tabuse M, Ohta S, Ohashi Y, Fukaya R, Misawa A, Yoshida K (2011). Functional analysis of HOXD9 in human gliomas and glioma cancer stem cells. Mol Cancer.

[9] Gaspar N, Marshall L, Perryman L, Bax DA, Little SE, Viana-Pereira M (2010). MGMT-independent temozolomide resistance in pediatric glioblastoma cells associated with a PI3-kinase-mediated HOX/stem cell gene signature. Cancer Res.

[10] Kola I, Landis J (2004). Can the pharmaceutical industry reduce attrition rates?. Nat Rev Drug Discov.

[11] Sleire L, Førde HE, Netland IA, Leiss L, Skeie BS, Enger PØ (2017). Drug repurposing in cancer. Pharmacol Res.

[12] Ekinci E, Rohondia S, Khan R, Dou QP (2019). Repurposing disulfiram as an anti-cancer agent: updated review on literature and patents. Recent Pat Anticancer Drug Discov.

[13] Skrott Z, Mistrik M, Andersen KK, Friis S, Majera D, Gursky J (2017). Alcohol-abuse drug disulfiram targets cancer via p97 segregase adaptor NPL4. Nature..

[14] Bax DA, Little SE, Gaspar N, Perryman L, Marshall L, Viana-Pereira M (2009). Molecular and phenotypic characterisation of paediatric glioma cell lines as models for preclinical drug development. PLoS ONE.

[15] Wang P, Lin C, Smith ER, Guo H, Sanderson BW, Wu M (2009). Global analysis of H3K4 methylation defines MLL family member targets and points to a role for MLL1-mediated H3K4 methylation in the regulation of transcriptional initiation by RNA polymerase II. Mol Cell Biol.

[16] Glaser S (2006). Multiple epigenetic maintenance factors implicated by the loss of Mll2 in mouse development. Development..

[17] Xu W, Su C-H, Lin IH, Tzeng T-Y, Hsieh W-T, Hsu M-T (2016). Regulation of IL-20 expression by estradiol through KMT2B-mediated epigenetic modification. PLoS ONE.

[18] Kerimoglu C, Agis-Balboa RC, Kranz A, Stilling R, Bahari-Javan S, Benito-Garagorri E (2013). Histone-methyltransferase MLL2 (KMT2B) is required for memory formation in mice. J Neurosci.

[19] Bhagwat AS, Vakoc CR (2015). Targeting transcription factors in cancer. Trends Cancer..

[20] Rheinbay E, Suvà Mario L, Gillespie Shawn M, Wakimoto H, Patel Anoop P, Shahid M (2013). An aberrant transcription factor network essential for wnt signaling and stem cell maintenance in glioblastoma. Cell Rep.

[21] Ligon KL, Alberta JA, Kho AT, Weiss J, Kwaan MR, Nutt CL (2004). The oligodendroglial lineage marker OLIG2 is universally expressed in diffuse gliomas. J Neuropathol Exp Neurol.

[22] Lu F, Chen Y, Zhao C, Wang H, He D, Xu L (2016). Olig2-dependent reciprocal shift in PDGF and EGF receptor signaling regulates tumor phenotype and mitotic growth in malignant glioma. Cancer Cell.

[23] Annovazzi L, Mellai M, Caldera V, Valente G, Schiffer D (2011). SOX2 expression and amplification in gliomas and glioma cell lines. Cancer Genomics Proteom.

[24] Eilers M, Eisenman RN (2008). Myc’s broad reach. Genes Dev.

[25] Blake JA, Ziman MR (2014). Pax genes: regulators of lineage specification and progenitor cell maintenance. Development..

[26] Uhlen M, Zhang C, Lee S, Sjostedt E, Fagerberg L, Bidkhori G, et al. A pathology atlas of the human cancer transcriptome. Science. 2017;357:eaan2507. 10.1126/science.aan2507. 10.1126/science.aan2507 28818916

[27] Cvek B (2012). Nonprofit drugs as the salvation of the world’s healthcare systems: the case of Antabuse (disulfiram). Drug Discov Today.

[28] Hothi P, Martins TJ, Chen L, Deleyrolle L, Yoon J-G, Reynolds B (2012). High-throughput chemical screens identify disulfiram as an inhibitor of human glioblastoma stem cells. Oncotarget..

[29] Triscott J, Lee C, Hu K, Fotovati A, Berns R, Pambid M (2012). Disulfiram, a drug widely used to control alcoholism, suppresses the self-renewal of glioblastoma and over-rides resistance to temozolomide. Oncotarget..

[30] Lun X, Wells JC, Grinshtein N, King JC, Hao X, Dang N-H (2016). Disulfiram when combined with copper enhances the therapeutic effects of temozolomide for the treatment of glioblastoma. Clin Cancer Res.

[31] Cantilena S, Gasparoli L, Pal D, Heidenreich O, Klusmann J-H, Martens JHA, et al. Non-toxic therapeutic direct targeting of MLL-fusion proteins using a rational drug screening approach (paper under submission). 2020.

[32] Veverka KA, Johnson KL, Mays DC, Lipsky JJ, Naylor S (1997). Inhibition of aldehyde dehydrogenase by disulfiram and its metabolite methyl diethylthiocarbamoyl-sulfoxide. Biochemical Pharmacol.

[33] Long Hannah K, Blackledge Neil P, Klose Robert J (2013). ZF-CxxC domain-containing proteins, CpG islands and the chromatin connection. Biochemical Soc Trans.

[34] Biaglow JE, Miller RA (2014). The thioredoxin reductase/thioredoxin system: Novel redox targets for cancer therapy. Cancer Biol Ther.

[35] Papaioannou M, Mylonas I, Kast RE, Brüning A (2013). Disulfiram/copper causes redox-related proteotoxicity and concomitant heat shock response in ovarian cancer cells that is augmented by auranofin-mediated thioredoxin inhibition. Oncoscience..

[36] Rao RC, Dou Y (2015). Hijacked in cancer: the KMT2 (MLL) family of methyltransferases. Nat Rev Cancer.

[37] Barski A, Cuddapah S, Cui K, Roh T-Y, Schones DE, Wang Z (2007). High-resolution profiling of histone methylations in the human genome. Cell..

[38] Liu P, Brown S, Goktug T, Channathodiyil P, Kannappan V, Hugnot JP (2012). Cytotoxic effect of disulfiram/copper on human glioblastoma cell lines and ALDH-positive cancer-stem-like cells. Br J Cancer.

[39] Ansari KI, Kasiri S, Mandal SS (2012). Histone methylase MLL1 has critical roles in tumor growth and angiogenesis and its knockdown suppresses tumor growth in vivo. Oncogene..

[40] Zhang C, Song C, Liu T, Tang R, Chen M, Gao F (2017). KMT2A promotes melanoma cell growth by targeting hTERT signaling pathway. Cell Death Dis.

[41] Lu C, Paschall AV, Shi H, Savage N, Waller JL, Sabbatini ME (2017). The MLL1-H3K4me3 axis-mediated PD-L1 expression and pancreatic cancer immune evasion. J Natl Cancer Inst.

[42] Ansari KI, Kasiri S, Mandal SS (2013). Histone methylase MLL1 has critical roles in tumor growth and angiogenesis and its knockdown suppresses tumor growth in vivo. Oncogene..

